# Risk factors, treatment, and outcome in dogs and cats with subdural hematoma and hemispheric collapse after ventriculoperitoneal shunting of congenital internal hydrocephalus

**DOI:** 10.1111/jvim.16861

**Published:** 2023-09-07

**Authors:** Daniela Farke, Anna K. Siwicka, Agnieszka Olszewska, Adriana Czerwik, Kathrin Büttner, Martin J. Schmidt

**Affiliations:** ^1^ Department of Veterinary Clinical Sciences, Small Animal Clinic Justus‐Liebig‐University, Frankfurter Strasse 114 35392 Giessen Germany; ^2^ Unit for Biomathematics and Data Processing, Faculty of Veterinary Medicine Justus Liebig‐University‐Giessen Giessen Germany

**Keywords:** canine, hemispheric collapse, hydrocephalus, magnetic resonance imaging, overshunting

## Abstract

**Background:**

Overshunting and hemispheric collapse are well‐known complications after ventriculoperitoneal shunt (VPS) implantation. Risk factors that predispose to overshunting, treatment options, and prognosis after therapeutic intervention have not been described.

**Hypothesis/Objectives:**

To identify preoperative risk factors for overshunting, the effect of surgical decompression, and their outcomes.

**Animals:**

Seventy‐five dogs and 7 cats.

**Methods:**

Retrospective case cohort study. Age, breed, sex, body weight, number of dilated ventricles, ventricle brain ratio, intraventricular pressure, and implanted pressure valve systems were evaluated as possible risk factors.

**Results:**

Overshunting had a prevalence of 18% (Cl 95% 9.9‐26.66). An increase of 0.05 in VBR increased the risk of overshunting by OR 2.23 (Cl 95% 1.4‐3.5; *P* = .001). Biventricular hydrocephalus had the highest risk for overshunting compared to a tri‐ (OR 2.48 with Cl 95% 0.5‐11.1) or tetraventricular hydrocephalus (OR 11.6 with Cl 95% 1.7‐81.1; *P* = .05). There was no influence regarding the use of gravitational vs differential pressure valves (*P* > .78). Overshunting resulted in hemispheric collapse, subdural hemorrhage, and peracute deterioration of neurological status in 15 animals. Subdural hematoma was removed in 8 dogs and 2 cats with prompt postoperative improvement of clinical signs.

**Conclusions and Clinical Importance:**

Biventricular hydrocephalus and increased VBR indicate a higher risk for overshunting. The use of differential valves with gravitational units has no influence on occurrence of overshunting related complications and outcomes. Decompressive surgery provides a favorable treatment option for hemispheric collapse and has a good outcome.

**Abbreviations**
ASD: antisiphoning devices
Cl: confidence level 95%
cm H_2_O: centimeters of water column
CSF: cerebrospinal fluid
FLAIR: fluid‐attenuated inversion recovery
ICP: intracranial pressure
IVP: intraventricular pressure
kg: kilogram
mm Hg: millimeters of mercury
MRI: magnetic resonance imaging
ms: milliseconds
PACS: Picture Archiving and Communication System
TE: echo time
TR: repetition time
VBR: ventricle brain ratio
VPS: ventriculoperitoneal shunting

## 1 INTRODUCTION

Internal communicating hydrocephalus is the most common malformation in dogs and cats.^1^, ^2^, ^3^, ^4^ Common clinical signs associated with ventricular enlargement and damage to the periventricular white matter are visual impairment, obtundation, ataxia, behavioral changes, and ventrolateral strabismus.^1^, ^2^, ^3^, ^4^, ^5^ Implantation of a ventriculoperitoneal shunt (VPS) system remains the treatment of choice, offering reconstitution of the brain parenchyma and resolution of clinical signs.^1^, ^2^, ^6^ However, among other complications, overshunting resulting in hemispheric collapse and subdural hemorrhage can occur in around 25% of cases during the first 3‐6 months after VPS implantation.^1^, ^2^ This complication is often fatal and leads to euthanasia in dogs and cats.

Overshunting is also a well‐known complication in human neurosurgery, which occurs in 10%‐18% of patients treated with VPS.^7^, ^8^ In children, posthemorrhagic hydrocephalus, aqueductus stenosis as well as VPS placement in the first months of life,^9^, ^10^, ^11^ neonatal meningitis,^11^, ^12^, ^13^ and implantation of low pressure opening valves are common risk factors.^9^, ^14^, ^15^ In adults, overshunting mostly occurs after changing from a lying into a standing position, when the hydrostatic pressure in the vertical catheter causes a siphon effect. Several valve technologies such as membrane antisiphoning devices, flow regulated valve systems, and adjustable gravitational valves are available for humans to reduce cerebrospinal fluid (CSF) flow across the valve and therefore, prevent a siphoning effect and overshunting.^16^, ^17^, ^18^, ^19^, ^20^ While risk factors for overshunting are well described and the benefit of gravitational valves and the efficacy of treatment options for hemispheric collapse were demonstrated in hydrocephalic humans,^21^, ^22^, ^23^, ^24^ such data are scarce in dogs and cats.^25^ The aim of this study was therefore first, to document risk factors for overshunting causing subdural hemorrhage and hemispheric collapse, and second to assess the efficacy of a decompressive surgery in shunt overdrainage and collapse.

## 2 MATERIALS AND METHODS

In this retrospective case cohort study, medical record databases of the Department of Veterinary Clinical Science, Small Animal Clinic, Justus‐Liebig‐University Giessen, Germany were searched for records with a diagnosis of internal hydrocephalus and implantation of a VPS dated from January 2001 to December 2021. Data collected from the records included the animals' age in months at the time of diagnosis, breed, sex, body weight, neutering status, clinical signs before and after VPS implantation, intraventricular pressure (IVP) measured in surgery, and implanted valve system (gravitational pressure valve vs prefixed differential pressure valves). Clinical signs related to overshunting, causing hemispheric collapse and subdural hemorrhage, as well as time of deterioration after VPS implantation were noted if they occurred. Outcomes for dogs and cats that underwent surgical decompression were evaluated based on the improvement of clinical signs. Clinical signs before VPS placement and 3 months after the second surgery were compared. Improvement of clinical signs is defined as resolution of 1 or more clinical signs that were present before VPS placement. Unchanged means same clinical signs before and after VPS treatment and second decompressive surgery. Euthanasia was performed on owners request, either because they declined further treatment or because of deterioration of clinical signs. Dead refers to a spontaneous death during treatment.

### 2.1 Magnetic resonance imaging

Imaging was performed using 3.0 Tesla high field MRI scanner (Phillips Intera Gyroscan, Philips Healthcare, Hamburg, Germany) or 1.5 Tesla high field MRI scanner (Siemens Verio, Siemens Healthcare, Erlangen, Germany). Images included at least sagittal, transverse, and dorsal T2‐weighted images (Turbo Spin Echo, TR 2900 ms, TE 120 ms, slice thickness 3 mm), and T1‐weighted pre‐ and post‐contrast medium administered images (TR 588, TE15, slice thickness 1 mm).

### 2.2 Image analysis

All MRI datasets were retrieved from the relevant PACS system and evaluated retrospectively by a board‐certified neurologist (DF). The images were evaluated for morphological and morphometric criteria allowing grading of ventricular enlargement and the presence of increased IVP as well as for the number of affected ventricles. These morphological criteria were the flattening of gyri and sulci, deformation of the interthalamic adhesion, disruption of the internal capsule, dilatation of the olfactory recess, and presence of periventricular edema. Corpus callosal height was measured and assessment of lateral ventricle size was performed using a ventricle/brain ratio (VBR) described elsewhere.^26^ Cases of hemispheric collapse after VPS implantation were evaluated for the location of subdural CSF and hemorrhage, the position of the ventricular catheter, and the presence of any brain herniation. A total collapse of the cerebral hemispheres was graded as “severe” collapse, a 50% compression of brain parenchyma by subdural fluid was graded as “moderate” and a 20% compression of brain parenchyma by subdural fluid was graded as “mild.”

Approval from the ethics committee of the Justus‐Liebig‐University was not sought as retrospective studies of images and records stored in the archive are not subject to ethical review.

### 2.3 Shunting procedures

VPS was performed using commercially available shunt systems (miniNAV and paediGav Miethke GmbH & Co KG, Potsdam, Germany).^27^ The shunt system in all dogs and cats included either a ball valve with prefixed opening pressures (miniNAV 5, 10, or 15 cm H_2_O) or a gravitational valve (paediGAV) that works in a position‐dependent manner. The gravitational valve system includes 2 ball valves; in a laying position, only the prefixed pressure unit offers resistance against the IVP (9 cm H_2_O = 6.6 mm Hg). In a standing position, the higher valve opening pressure of the gravitational unit (19 cm H_2_O = 14 mm Hg) must be overcome, which offers additional protection against overshunting. Both systems have a CSF prechamber included that was placed subcutaneously at the level of the cervical spine. There was no rationale for the use of 1 or the other system. There was a trend to use gravitational valves in the first 10 years of the observation period, whereas valves with prefixed opening pressures prevailed in the second decade. Opening pressures of the prefixed differential valve systems were chosen in relation to the measured IVP in the individual animals.

IVP was measured using a commercially available system for humans using a single use piezo‐resistive strain‐gauge sensor mounted in a miniature titanium case at the tip of a flexible nylon catheter (MicroSensor ICP probe) as described previously.^28^

IVP was measured in 47 animals, all of them received a prefixed differential pressure valve. Dogs with IVP < 5 mm Hg received a valve with an opening pressure of 5 cm H_2_O, in dogs with IVP between 6 and 12 mm Hg valves with an opening pressure of 10 cm H_2_O was chosen. In all other dogs (IVP > 13 mm Hg), 15 cm H_2_O valves were used. In animals that received a gravitational valve system IVP was not measured.

### 2.4 Statistical analysis

Statistical analysis was performed using a commercial statistical software package (Base SAS 9.4 Procedures Guide: Statistical Procedures, 2nd edition ed. Statistical Analysis System Institute Inc., Cary, North Carolina, USA). Prevalence of hemispheric collapse was assessed and a mean value and SD were calculated to determine a binominal proportion confidence level (Cl) of 95%. Overshunting related complications were evaluated as a dependent, dichotomous variable and are defined as subdural hemorrhage and hemispheric collapse. Breed, body weight, age, VBR, IVP, number of affected ventricles, implanted valve system and pressure settings within differential pressure valves were evaluated as independent variables to evaluate their influence on overshunting related complications. Because of a low sample size logistic regression analysis was performed for every single variable.

A log10 transformation was performed on the variables age and body weight and to gain normally distributed data and a Wald chi‐square test were performed to compare these variables in animals with and without overshunting related complications. Sex distribution was further summarized to male and female because of very few neutered animals and an exact Pearson chi‐square test and logistic regression analysis was performed to evaluate the sex as a risk factor for overshunting related complications. Species correlation to overshunting related complications was done by using an exact Pearson chi‐square test and logistic regression analysis. The evaluation of breed as a risk factor for overshunting was not possible for every single breed because of distribution of data. Therefore, breeds were grouped into small breeds (<5 kg), medium breeds (6‐20 kg), and large breeds (<21 kg) and an exact Pearson chi‐square test and logistic regression analysis was used to evaluate a correlation between these groups and overshunting related complications. The number of affected ventricles and VBR were normally distributed. An exact Pearson chi‐square test and a logistic regression based on a maximum likelihood estimation were performed to evaluate the correlation of a bi‐, tri, or tetraventricular hydrocephalus in animals with and without overshunting related complications. Furthermore, an odds ratio calculation was performed to compare the likelihood of overshunting related complications between groups with different affected ventricles. A Wald chi‐square test and an odds ratio were performed on VBR to compare between animals with and without overshunting related complications. A log10 transformation was performed on the data of IVP to set a normal data distribution and a Wald chi‐square test was used to evaluate correlation of IVP to overshunting related complications. To evaluate the use of different valve systems (gravitational vs prefixed differential pressure valves) an exact Pearson chi‐square test was used to look for dependencies between animals with and without overshunting related complications. To evaluate the influence of different pressure settings (5, 10, and 15 cm H_2_O) of the prefixed differential pressure valves on the development of overshunting related complications another exact Pearson chi‐square test was performed. For all statistical tests, a significance level of 0.05 was applied.

## 3 RESULTS

### 3.1 Animals

Eighty‐two animals (75 dogs and 7 cats) were included in the study. The median age was 7 months (1‐67 months), median body weight was 3.7 kg (1.1‐58 kg). There were 26 females, 2 neutered females, 43 males, and 11 neutered males. Fifteen animals presented with a hemispheric collapse after VPS. Time of associated deterioration of neurologic status ranged from 4 to 60 days after VPS (median 18 days). Hemispheric collapse and associated subdural hemorrhage had a prevalence of 18% (n = 15 animals; Cl 95% 9.9‐26.66).

### 3.2 Risk factor evaluation

All animals were evaluated for species, body weight, age, sex, number of affected ventricles. No significant influences of body weight, age, sex, or breed were found (Table 1). VBR was calculated in all, but 1 animal. A higher VBR was significantly associated with a higher risk for overshunting related complications. An increase of 0.05 in VBR increased the risk of overshunting by OR 2.23 (Cl 95% 1.4‐3.5; *P* = .001; Table 1). Furthermore, the number of affected ventricles was also a significant parameter for overshunting (*P* = .05). A biventricular hydrocephalus carried a higher risk of overshunting related complications compared to a tri‐ (OR 2.48 with Cl 95% 0.6‐11.1) or tetraventricular hydrocephalus (OR 11.6 with Cl 95% 1.7‐81.1). Triventricular hydrocephalus in turn was also at higher risk for overshunting (OR 4.68 Cl 95% 0.9‐23.2) compared to tetraventricular hydrocephalus (Table 1). IVP was measured in 47 cases. IVPs ranged from 1 to 35 mm Hg. A higher or lower IVP was not identified as a risk factor for overshunting (Table 1). Twenty‐eight gravitational pressure valves and 48 differential pressure valves were used; for 6 animals, the implanted valve system was not recorded. Within the group that was affected by hemispheric collapse and subdural hemorrhage 6 animals received a gravitational valve and 8 received a prefixed differential pressure valve, for 1 animal the valve system was not recorded. The type of valve system had no influence on the development of subdural hematoma or hemispheric collapse (Table 1). Furthermore, there was no difference observed in the use of high or low prefixed differential pressure valve systems (Table 1). Species, however, revealed a significant influence on the occurrence of overshunting. All cats in this study presented with a VBR > 0.81 and were more likely to develop hemispheric collapse and subarachnoid hemorrhage than dogs (OR 13.13 Cl 95% 1.8‐145.8; *P* = .004; Table 1).

**TABLE 1:** Summary of statistical results of the logistic regression analysis showing number of observations used, results of Wald chi‐square test, *P*‐value, and odds ratio with associated confidence level of 95% for each tested variable.

Tested variable	Nr. of observations used	Wald chi‐square	*P*‐value		Odds ratio	Confidence level 95%
Log10 (weight)	82	0.011	.92		0.94	0.29‐3.03
Log10 (age)	82	0.13	.72		0.79	0.23‐2.78
Sex	82	0.22	.78	Male vs female	1.31	0.41‐4.2
Breed						
Small (<5 kg)	82	0.66	.72	Small vs medium	1.51	0.35‐6.47
Medium (5‐20 kg)				Small vs large	0.8	0.24‐2.68
Large (>20 kg)				Medium vs large	0.53	0.11‐2.46
Species						
Dogs	82	8.29	.004	Cats vs Dogs	13.13	1.8‐145.78
Cats						
VBR	81	11.98	.001		2.23	1.42‐3.5
Affected ventricles						
Biventricular	81	6.22	.05	Bi‐ vs Triventric.	2.48	0.56‐11.07
Triventricular				Bi‐ vs Tetravent.	11.6	1.66‐81.10
Tetraventricular				Tri‐ vs Tetravent.	4.68	0.94‐23.18
VPS system						
Gravitational valve	76	0.09	.75	Gravitational vs prefixed differential pressure valve	0.83	0.25‐2.72
Prefixed differential pressure valve						
Prefixed differential pressure valves						
Opening pressures	48	1.19	.55	5 vs 10 cm H_2_O	1.42	0.34‐6.37
				5 vs 15 cm H_2_O	0.29	0.02‐5.5
				10 vs 15 cm H_2_O	0.2	0.01‐3.91
Log10 (IVP)	46	2.86	.09		0.17	0.021‐1.33

*Note*: A log10 transformation was used for weight, age, IVP, and VBR.

Abbreviations: IVP, intraventricular pressure; VBR, ventricle brain ratio.

### 3.3 Outcome of subdural hematoma excavation

**FIGURE 1:**
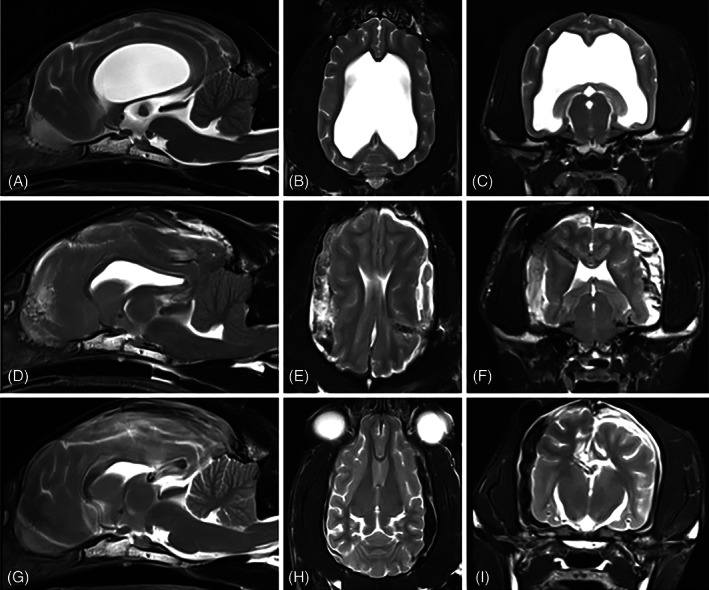
T2 weighted MRI pictures of a 49 months‐old Italian greyhound before ventriculoperitoneal shunt (VPS) placement (A‐C), 4 days after VPS placement with hemispheric collapse (D‐F), and 3 months after bilateral craniotomy and suboccipital craniectomy (G‐I). Note the concave contour of the ventricles at time of hemispheric collapse and the subarachnoid accumulation of T2 hyper‐ and hypointense material which compresses both cerebral hemispheres (D‐F). The ventricular catheter is completely surrounded by brain tissue (E, F). There is severe cerebellar herniation through the foramen magnum (D). At 3 months control (G‐I) there is still some hypointense lining following the left parietal bone (H, I), but the ventricular catheter is positioned within the ventricle (G, l) and there is no evidence for cerebellar herniation (G).

Eight dogs and 2 cats underwent decompressive surgery. Corresponding to the found risk factor, the VBR before VPS implantation was >0.85 in all but 1 case. All animals received a bilateral craniotomy at the level of the left and right parietal bone to drain subdural CSF and hemorrhage. The amount of evacuated hemorrhagic fluid from the subdural space was a median of 15 mL but ranged from 2 to 65 mL. Four animals received additional suboccipital craniectomy because of foraminal herniation visible on previous MRI (Figure 1). The ventricular catheter was still within the ventricle in 6/10 animals, but the cortical mantle was wrapped around the tip of the catheter in these cases. The ventricular catheter was within the brain parenchyma in 2/10 animals and outside the ventricle within the subarachnoid space in 1/10 animals. For 1 animal, the position of the ventricular catheter was not documented. The ventricular catheter was left in place in all animals and ventricular catheter position was documented. The majority of animals showed an improvement of clinical signs 1 to 3 days after surgical decompression (8/10). Overall, clinical signs improved after surgical decompression. Clinical presentation before and after surgery is presented in Table S1. Some clinical signs like strabismus (1/1), nystagmus (1/1), and seizures (4/4), however, remained but were also present even before VPS placement. Additional therapy included pain medication like methadone 0.1 mg/kg i.v. every 4 h (10/10), metamizole 50 mg/kg i.v. every 8 h (8/10), metacam 0.1 mg/kg i.v. every 24 h (2/10). Antibiotic therapy included cefotaxime 20 mg/kg i.v. every 8 h (10/10). Antiemetic therapy was required in 3/10 cases (metoclopramide 0.1 mg/kg i.v.). Antiepileptic treatment during hospitalization included phenobarbital at varying doses but at a minimum of 2.5 mg/kg i.v./p.o. every 12 h (4/10), midazolam constant rate infusion 0.2 mg/kg/h i.v. (2/10) and levetiracetam 20 mg/kg i.v. every 8 h (1/10). After dehospitalization seizures were under control with phenobarbital at varying doses but at a minimum of 2.5 mg/kg every 12 h. One dog (Samoyed) remained unchanged and showed visual deficits, ataxia, and strabismus. One European Shorthair cat was euthanized because of worsening of clinical signs 5 days after decompressive surgery.

## 4 DISCUSSION

VPS is recommended as the treatment of choice for dogs and cats with internal hydrocephalus.^1^, ^2^, ^6^ Despite good long‐term outcomes in most dogs and cats, the procedure continues to be associated with well‐known complications. After volume decrease of the ventricles and reduction of the IVP, the subarachnoid and subdural spaces expand, often beyond physiological limits. CSF accumulates in this space creating a hygroma, which is usually clinically irrelevant. In some cases, expansion of the subarachnoid space results in rupture of the bridging veins and subdural or subarachnoid hematoma formation. The mixture of blood and CSF then results in a hemorrhagic subdural fluid that causes compression of the hemispheres and hemispheric collapse. This complication occurs after VPS in animals and humans alike.^1^, ^29^, ^30^, ^31^ Prevalence of overshunting related complications such as hemispheric collapse and subdural hematoma was 18% (Cl 95% 9.9‐26.66) among the animals treated with VPS in this study. This is similar to other reported complication rates in veterinary and human medicine.^1^, ^2^, ^7^, ^8^

The major predisposing factor in humans is the implantation of a VPS in very young infants in the early postnatal phase.^9^, ^10^, ^11^ The high prevalence of subdural effusions in infants is likely because of the fact that their brain parenchyma has less stiffness and yields more easily after a decrease of IVP resulting in rupture of the bridging veins in the subarachnoid space.^32^, ^33^ We could not document age as a risk factor, however, whereas human infants often undergo surgery in their first days of life, dogs and cats are usually not operated on during their neonatal phase. The subgroup most at risk in humans is therefore not included in our study group.

The potential influence of parenchymal stability on the occurrence of hemispheric collapse and subdural hematoma might be supported by the fact that severe ventricular enlargement or increased VBR was associated with increased risk of hematoma formation and hemispheric collapse in this study. This study found a higher risk for cats to develop overshunting compared to dogs, but this is surely biased by the fact that the cats in this study all had high VBRs (>0.81). Chronicity of severe ventricular enlargement was also found to be predisposing for hemispheric collapse and subdural hematoma formation in children.^9^, ^34^, ^35^, ^36^

Another predisposing risk factor for subdural hematoma and hemispheric collapse in humans is the development of internal hydrocephalus as a consequence of periventricular/intraventricular hemorrhage. The finding of posthemorrhagic hydrocephalus as a risk factor for subdural hemorrhage might again be associated with the young age of infants as posthemorrhagic hydrocephalus most frequently occurs in preterm infants. This finding is, however, controversial as some studies did not find an association between the etiology of hydrocephalus and occurrence of overshunting and subdural hemorrhage.^37^ While the development of hydrocephalus after intraventricular injection of autologous blood in dogs is clearly demonstrated,^38^, ^39^ the prevalence of naturally occurring periventricular hemorrhage as an underlying cause for internal hydrocephalus is unknown.^40^ We could only find evidence of hemorrhage in one 2‐month‐old dachshund in our study group, which again prevents a comparison between human patients and our study group. The systematic use of susceptibility‐weighted studies aiming to identify periventricular hemorrhage in hydrocephalic animals would be valuable to evaluate this finding as a risk factor in the future. The relevance of this finding lies in the fact that intraventricular thrombolytic treatment is recommended as treatment for children with posthemorrhagic hydrocephalus,^41^ which might be an alternative or adjunct therapy for animals, too.

Evaluation of IVP dynamics clearly demonstrated the influence of body position on CSF drainage in hydrocephalic human adults.^42^, ^43^, ^44^, ^45^, ^46^, ^47^ Higher CSF draining was associated with a standing position and the addition of the hydrostatic pressure in the shunt system to IVP that in sum act on the valve's resistance to CSF flow.^48^, ^49^ The use of shunts without antisiphoning devices is another major risk factor for overdrainage and hematoma in most human studies.^50^ We could not find a significant difference in the use of gravitational or prefixed differential pressure valves with regard to the occurrence of overshunting related complications. The impact of the hydrostatic force within the shunt depends on the distance between the intraventricular and peritoneal cavities.^7^ This distance is much lower in dogs, especially in toy‐breed dogs and cats, which could explain the missing benefit for differential valves in animals. The positioning of the gravitational valve system might play an important role as it affects the function of CSF drainage in the standing human where it is placed in a parallel line to the vertebral column. Dogs in this study received the gravitational valves in a vertical line to the vertebral column in order to take care of the different position during standing compared to humans. Due to changes of subcutaneous valve positioning during growth, the position sometimes changes to a more oblique manner. The effect of gravitational valve positioning and the development of overshunting related complications in humans or animals remains to be investigated. Occurrence of overshunting was also no more frequent in larger dogs, which poses the question as to whether gravitational forces have a relevant impact on CSF drainage in dogs. The benefit of a gravitational valve to avoid posture‐dependent overdrainage is not consistently documented in humans.^50^ It is suggested that it was not the pressure changes under continuous drainage that resulted in subdural hemorrhage, but rather the sudden drop of IVP after insertion of the ventricular catheter through the cortical mantle. In favor of this theory is the finding that subdural hemorrhage can also occur after endoscopic third ventriculostomy, in which a siphoning effect can be ruled out.^51^, ^52^

Sudden increase in IVP might enhance CSF drainage independent from gravitational forces inside the shunt. In humans that forced respiration,^53^ increased abdominal pressure,^54^ and increased resistance of venous outflow increase intracranial pressure (ICP),^55^ which exerts an influence on forced CSF pulsation, as in dogs.^56^ ICP measurements in clinically healthy Beagles have significant variation from baseline ICP accompanying physical activity and, especially a head down posture increasing ICP to >25 mm Hg,^57^ which is far beyond the opening pressures of the valves used here. The prefixed pressure valve systems used in this study were chosen with pressure settings of 5, 10, and 15 cm H_2_O, to ensure a physiological IVP of 5‐12 mm Hg after VPS placement. However dynamic IVP changes with physical activity might play a relevant role in the pathophysiology of hemispheric collapse in animals.

The number of involved cerebral ventricles had a clear effect on the occurrence of ventricular collapse, with biventricular and triventricular hydrocephalus having a higher risk. A potential explanation could be found in intraventricular CSF flow dynamics. During cardiac systole, the brain fills with blood, increasing the parenchymal volume and compressing the cerebral ventricles. This is the driving force of CSF that flows from the lateral cerebral ventricles through the third ventricle, mesencephalic aqueduct, and fourth ventricle into the subarachnoid space. This flow is not unidirectional, but rather back and forth with a net caudad flow of CSF out of the ventricles.^58^ If the mesencephalic aqueduct is patent, reflow of CSF from the fourth into the third and lateral ventricle might compensate for temporary excessive CSF drainage through the shunt and support the cortical mantle. Children with aqueductal stenosis have an increased risk for hemispheric collapse.^9^ Reflow from the fourth ventricle is blocked in these patients, which might confirm the relevance of reflow from the fourth ventricle for support of the cerebral mantle. In our study, 8 of 15 affected animals were presented with triventricular hydrocephalus, aqueductal stenosis was only present in 2 of these animals.

Given the retrospective character of our study and the single logistic regression analysis of each variable we cannot exclude bias. One example is that our results show a higher risk for cats to develop overshunting compared to dogs which is biased by the fact that the cats in this study all had high VBRs (>0.81). Influences from 1 variable to the other cannot be excluded completely for the other risk factor evaluations as well.

Ten animals, 8 dogs and 2 cats, underwent bilateral craniotomy and evacuation of hemorrhagic fluid (subdural CSF and hemorrhage) for decompression. Another 4 dogs received additional suboccipital craniectomy because of foraminal herniation visible on previous MRI. The position of the ventricular catheter was within the lateral ventricle (9/15), or within the parenchyma in 4/15 animals. In 1 animal, the ventricular catheter was outside the ventricle and brain parenchyma and in 1 animal the location of the ventricular catheter was not reported. But even if the ventricular catheter was within the ventricle the cortical mantle was wrapped around the tip of the catheter because of the hemispheric collapse, so that the ventricular catheter became obstructed. During the surgical procedure the ventricular catheter was left in place and was not further manipulated. Therefore, a cortical rebuilding was only achieved by surgical decompression, probably because the ventricular catheter was blocked by the collapsed cortical mantle in most cases until CSF was reproduced within the ventricles. Within 5 days after the surgical procedure a refilling of the prechamber was noticed in 9/10 animals, indicating CFS flow through the ventricular catheter into the VPS.

Animals that experienced hemispheric collapse had a time of deterioration after VPS implantation of median 18 days (4‐60 days) which is less time than reported for overall complications.^1^, ^2^ This might indicate that hemispheric collapse in dogs and cats is an early complication compared to obstruction and shunt infection or displacement. Shunt displacement might be associated with growth of the animal in cases of shunt systems that are not placed in loops and become too short. Obstruction on the other hand is because of the production of protein and inflammatory cells within the CSF. Both conditions might need more time to develop than overshunting related complications such as hemispheric collapse.

Clinical signs at deterioration included, obtundation stupor or coma (12/15), ataxia/tetraparesis (10/15), vision deficits (7/15), seizures (5/15), pain (4/15), strabismus (4/15), absent physiological nystagmus (3/15), nystagmus (3/15), and 1 dog showed a Cushing's reflex as a sign of increased ICP (1/15). These signs are highly indicative not only of forebrain but also of brainstem involvement. The presence of Cushing's reflex (hypertension, bradycardia, and irregular breathing pattern) furthermore indicates an abrupt increase in ICP, and immediate intervention should follow. This patient was an Italian greyhound who showed clinical deterioration 4 days after VPS placement during hospitalization, so that MRI and decompressive surgery including suboccipital craniectomy took place immediately. The dog recovered well from the procedure and showed a normal neurological examination and good quality of life at a control examination 3 months after surgical decompression.

Clinical signs such as nystagmus, seizures, and strabismus were present in some dogs before placement of VPS and remained throughout therapy. The occurrence of seizures in this study seems to be higher than the reported prevalence of 1.7% for hydrocephalus in animals.^59^ However, as we only included animals that underwent surgery it does not reflect the overall population of animals presenting with internal hydrocephalus. Seizures were under well control with phenobarbital at various doses in 3/4 animals. One cat that also suffered from seizures was euthanized because of a comatose mental state and ongoing generalized seizures 5 days after decompressive surgery. The overall outcome for dogs and cats that received decompressive surgery after hemispheric collapse was favorable with 8/10 animals surviving and showing improvement of clinical signs. The lack of an untreated or medically treated control group remains a limitation of this study and given the low number of surgically treated cases in this study, further investigation is needed to provide more evidence and prognostic information in these cases.

## CONFLICT OF INTEREST DECLARATION

Authors declare no conflict of interest.

## OFF‐LABEL ANTIMICROBIAL DECLARATION

Authors declare no off‐label use of antimicrobials.

## INSTITUTIONAL ANIMAL CARE AND USE COMMITTEE (IACUC) OR OTHER APPROVAL DECLARATION

Authors declare no IACUC or other approval was needed.

## HUMAN ETHICS APPROVAL DECLARATION

Authors declare human ethics approval was not needed for this study.

## Supporting information


**Table S1:** Summary of animals that experienced hemispheric collapse and received decompressive surgery. Listed are breed, age at presentation in months and implantation of ventriculoperitoneal shunt (VPS) and clinical signs at presentation. Magnetic resonance imaging (MRI) findings include the Nr. Vent = number of affected ventricles (2 = lateral ventricles, 3 = lateral and third ventricles. 4 = lateral, third and fourth venhicle, (A) = aqueductal stenosis) and ventricle brain ratio (VBR). IVP (intraventricular pressure) refers to the intraoperative measurements at time of VPS placement and the valve system used in surgery is further specified as G = gravitational system, or differential pressure system at various opening pressure (5, 10, or 15 cm H_2_O). Time of deterioration is given in days after VPS placement and clinical signs are described. MRI signs at deterioration are further evaluated for grade of hemispheric collapse (HC) (Severe HC = complete collapse of brain parenchyma, moderate HC = 50% compression of brain parenchyma, mild HC = 20% compression of brain parenchyma) and presence of herniation (foraminal herniation and caudal transtentorial herniation) and position of the ventricular catheter within the ventricular system, or outside the brain parenchyma. The type of surgery performed is described as BC = bilateral craniotomy and SC = suboccipital craniectomy. NP = not performed. The amount of evacuated subdural fluid is given in ml. Time of hospitalization after the surgical decompression of the hemispheric collapse is given in days. Clinical signs after decompressive surgery are given during hospitalization (including the status of the CSF prechamter. if recorded) and 3 months after surgery. The outcome summarizes the clinical condition 3 months after decompressive surgery compared to signs before VPS placement. Asterisks = missing data.
