# Supplementary material for: Risk factors, treatment, and outcome in dogs and cats with subdural hematoma and hemispheric collapse after ventriculoperitoneal shunting of congenital internal hydrocephalus
Source: J Vet Intern Med. 2023 Sep 7;37(6):2269–77. doi: 10.1111/jvim.16861 (PMC10658535; doi:10.1111/jvim.16861)
Supplement: Supplementary file 1 — Table S1: Summary of animals that experienced hemispheric collapse and received decompressive surgery. Listed are breed, age at presentation in months and implantation of ventriculoperitoneal shunt (VPS) and clinical signs at presentation. Magnetic resonance imaging (MRI) findings include the Nr. Vent = number of affected ventricles (2 = lateral ventricles, 3 = lateral and third ventricles. 4 = lateral, third and fourth venhicle, (A) = aqueductal stenosis) and ventricle brain ratio (VBR). IVP (intraventricular pressure) refers to the intraoperative measurements at time of VPS placement and the valve system used in surgery is further specified as G = gravitational system, or differential pressure system at various opening pressure (5, 10, or 15 cm H2O). Time of deterioration is given in days after VPS placement and clinical signs are described. MRI signs at deterioration are further evaluated for grade of hemispheric collapse (HC) (Severe HC = complete collapse of brain parenchyma, moderate HC = 50% compression of brain parenchyma, mild HC = 20% compression of brain parenchyma) and presence of herniation (foraminal herniation and caudal transtentorial herniation) and position of the ventricular catheter within the ventricular system, or outside the brain parenchyma. The type of surgery performed is described as BC = bilateral craniotomy and SC = suboccipital craniectomy. NP = not performed. The amount of evacuated subdural fluid is given in ml. Time of hospitalization after the surgical decompression of the hemispheric collapse is given in days. Clinical signs after decompressive surgery are given during hospitalization (including the status of the CSF prechamter. if recorded) and 3 months after surgery. The outcome summarizes the clinical condition 3 months after decompressive surgery compared to signs before VPS placement. Asterisks = missing data. [file JVIM-37-2269-s001.pdf]

Nr.	Breed	Age months	Clinical signs before VPS	Nr. Vent.	VBR	IVP mm Hg	Valve system	Time of deterio-ration days	Clinical signs at deterioration and CSF prechamber status	Grade of collapse and presence of herniation	Ventricular catheter position	Surgery	Subdural fluid ml	Time of hospital-isation (days)	Clinical signs during hospitalisation and CSF- prechamber status	Clinical signs 3months after decompressive surgery	Outcome
1	European shorthair cat	6	Abnormal skull conformation, ataxia, obtundation, strabismus, pain, reduced physiological nystagmus	3	0.95	6	G	5	Abnormal skull conformation, ataxia, obtundation, focal seizures, pain, strabismus, absent physiological nystagmus, no refilling of CSF prechamber	Severe HC Foraminal H.	Outside ventricle	BC	54	2	Stupor, focal/ generalized seizures, absent physiological nystagmus and cranial nerve reflexes. No refilling of CSF prechamber	*	Eutha
2	Labrador retriever	2.5	Abnormal skull conformation, Strabismus, obtundation	2	0.85	7	10 cm H_2O	10	Abnormal skull conformation, non ambulatory tetraparesis, obtundation, disorientation, strabismus, reduced physiological nystagmus, no refilling of CSFprechamber	Severe HC Foraminal H. C. transtentorial H.	Within ventricle	BC+SC	65	6	2 days post OP normal mentation, ambulatory, normal physiological nystagmus, improved strabismus. Refilling of CSF prechamber	No deficits	Improved
3	Italian greyhound	49	Ataxia, obtundation, pain	2	0.69	5	5 cm H_2O	4	Coma, non ambulatory tetraparesis, absent menace, absent physiological nystagmus, bilateral mydriasis, absent cranial nerve reflexes and PLR, Cushing response, no refilling of CSF prechamber	Moderate HC Foraminal H.	Within brain parenchyma	BC+SC	12	8	2 days post OP normal mentation, ambulatory, normal physiological nystagmus, normal menace and PLR. Refilling of CSF prechamber	No deficits	Improved
4	Golden retriever	1.5	*	*	*	*	*	*	Abnormal skull confirmation, ataxia, obtundation, blind, disorientation, strabismus, nystagmus, pain	Severe HC Foraminal H. C. transtentorial H.	*	BC+SC	60	4	1 day post OP improved mentation and gait, no nystagmus, less painful, reduced menace response. CSF prechamber not recorded	No deficits	Improved
5	European shorthair cat	11	Abnormal skull conformation, obtundation, seizures	2	0.87	*	10 cm H_2O	46	Abnormal skull conformation, ataxia, obtundation, pain, vision impairment, increased extensor tone, bilateral miosis, nystagmus, seizures, no refilling of CSF prechamber	Moderate HC Foraminal H.	Within ventricle	BC+SC	2	6	3 days post OP Improved mentation and gait, no nystagmus, reduced menace response. Refilling of CSF prechamber	No deficits, but seizures,	Improved
6	German shepherd	9	Obtundation, non ambulatory tetraparesis, seizures, pain, nystagmus, vision impairment, impaired learning ability	3 (A)	0.89	20	15 cm H_2O	31	Ataxia, circling, blind, disorientation, seizures, no refilling of CSF prechamber	Moderate HC	Within ventricle	BC	35	3	3 days post OP no disorientation, no circling. No refilling of CSF prechamber	seizures, reduced menace response. Refilling of CSF prechamber	Improved
7	Chihuahua	8	Obtundation, vision impairment	4	0.86	12	10 cm H_2O	28	Obtundation, blind, disorientation, pain, no refilling of CSF prechamber	Moderate HC	Within brain parenchyma	BC	9	7	3 days post OP normal mentation, improved vision, less painful. Refilling of CSF prechamber	No deficits	Improved
8	Samojede	8	Obtundation, Blind, circling, nystagmus, strabismus	3 (A)	0.91	1	5 cm H_2O	19	Ataxia, obtundation, circling, blind, nystagmus, no refilling of CSF prechamber	Severe HC C. transtentorial H.	Within ventricle	BC	9	7	7 days post OP mild improvement of gait, unchanged blindness, nystagmus and circling. Refilling of CSF prechamber	blind, occasional circling	Un-changed
9	Labrador retriever	6	Ataxia, obtundation, vision impairment	3	0.91	3	5 cm H_2O	19	Ataxia, circling, painful, reduced nasal sensation, vision impairment, no refilling of CSF prechamber	Severe HC	Within ventricle	BC	15	4	1 day post OP improved gait, no circling Refilling of CSF prechamber	No deficits	Improved
10	English bulldog	23	Ataxia, obtundation, vision impairment, seizures	3	0.86	*	G	60	Obtundation, Cluster seizures, no refilling of CSF prechamber	Moderate HC	Within ventricle	BC	50	5	3 days post OP improved mentation. Refilling of CSF prechamber	No deficits, but seizures	Improved
11	Rag doll	10	Non ambulatory tetraparesis, obtundation, vision impairment, strabismus	3	0.94	*	G	17	Stupor, seizures, no refilling of CSF prechamber	Severe HC	Within brain parenchyma	NP	*	*	*	*	Eutha
12	European shorthair cat	3	Abnormal skull conformation, ataxia	4	0.98	*	G	5	Obtundation, non ambulatory tetraparesis, no refilling of CSF prechamber	Moderate HC	Within ventricle	NP	*	*	*	*	Eutha
13	British shorthair cat	1	Abnormal skull conformation, ataxia, blind, strabismus, impaired learning ability	2	0.94	*	G	6	Sudden death	Severe HC Foraminal H. C. transtentorial H.	Within ventricle	NP	*	*	*	*	Dead
14	West Highland White Terrier	3	Abnormal skull conformation, ataxia, obtundation, vision impairment, pain, strabismus	3	0.88	*	G	7	Ataxia, obtundation, circling, strabismus, no refilling of CSF prechamber	Severe HC C. transtentorial H.	Within brain parenchyma	NP	*	*	*	*	Eutha
15	Yorkshire Terrier	22	Ataxia, blind, pain	3	0.89	15	15 cm H_2O	15	Ataxia, blind, anorexia, aggressive behavior, no refilling of CSF prechamber	Moderate HC	Within ventricle	NP	*	*	*	Blind	Improved

Supplementary Table 1: Summary of animals that experienced hemispheric collapse and received decompressive surgery. Listed are breed, age at presentation in months and implantation of ventriculoperitoneal shunt (VPS) and clinical signs at presentation. Magnetic resonance imaging (MRI) findings include the Nr. Vent = number of affected ventricles (2 = lateral ventricles, 3 = lateral and third ventricles, 4 = lateral, third and fourth ventricle, (A) = aqueductal stenosis) and ventricle brain ratio (= VBR). IVP (= intraventricular pressure) refers to the intraoperative measurements at time of VPS placement and the valve system used in surgery is further specified as G = gravitational system, or differential pressure system at various opening pressure (5, 10, or 15 cm H_2O). Time of deterioration is given in days after VPS placement and clinical signs are described. MRI signs at deterioration are further evaluated for grade of hemispheric collapse (HC) (Severe HC = complete collapse of brain parenchyma, moderate HC= 50% compression of brain parenchyma, mild HC= 20% compression of brain parenchyma) and presence of herniation (foraminal herniation and caudal transtentorial herniation) and position of the ventricular catheter within the ventricular system, or outside the brain parenchyma. The type of surgery performed is described as BC= bilateral craniotomy and SC= suboccipital craniectomy, NP = not performed. The amount of evacuated subdural fluid is given in ml. Time of hospitalisation after the surgical decompression of the hemispheric collapse is given in days. Clinical signs after decompressive surgery are given during hospitalisation (including the status of the CSF prechamber, if recorded) and 3 months after surgery. The outcome summarizes the clinical condition 3 months after decompressive surgery compared to signs before VPS placement. Asterisks = missing data.
